# Anterior minimal invasive internal fixator versus open plating in treatment of unstable pelvic ring injuries

**DOI:** 10.1007/s00402-025-05891-z

**Published:** 2025-05-09

**Authors:** Khaled Omran, Ahmed Nady, Ahmed Hisham Abdelhaliem Ali, Mohamed Sayed Khamies

**Affiliations:** Department of Orthopedic Surgery and Traumatology, Minia University Hospital, Faculty Of Medicine, Minia University, Minya, Egypt

**Keywords:** Anterior minimal invasive internal fixator (INFIX), Pelvic ring injuries, Open plating

## Abstract

**Background:**

Pelvic fractures are frequently presented to major trauma centers and are mostly associated with high morbidity, especially in polytrauma patients. While stable fractures are managed non-operatively in most cases, unstable patterns necessitate surgical fixation because of the significant morbidity and mortality related to unstable pelvic fractures.

**Objective:**

To compare the clinical, radiological, and functional outcomes in patients with unstable pelvic ring fractures treated with anterior minimal invasive internal fixator (INFIX) versus plate fixation.

**Study design:**

Retrospective single-center clinical study.

**Methods:**

This research was conducted on 60 patients with unstable pelvic ring injuries. There were 38 (63.3%) males and 22 (36.7%) females with a mean age of 33.57 ± 11.03 years. Half of the patients were managed with INFIX, and the other half with open plating. Pelvic deformity index (PDI) and symphyseal widening were used to assess the pelvic ring reductions.

**Results:**

Reduction of symphyseal widening was significantly better in open plating than in the INFIX group (*P* < 0.001), but both techniques are comparable in reducing PDI. Time to surgery, whole operative time, anterior pelvic ring procedure time, hospital stay, and blood loss decreased significantly in the INFIX group compared to the plate group (*P* < 0.001).

**Conclusion:**

INFIX is a minimally invasive procedure that provides much lower operative time and less blood loss than open plating in the anterior pelvic ring fracture management. Meanwhile, more anatomical reduction of the anterior pelvic ring fracture was achieved through plate fixation. There was a significant difference in the postoperative symphyseal diastasis achieved with plating compared to INFIX [plating (5.47 ± 2.03 mm) lower than INFIX(11.40 ± 3.76 mm)].

## Introduction

Unstable pelvic fractures are substantial injuries that primarily result from high-impact trauma and can result in a range of consequences. They represent around 1.5-3% of all bone injuries, with a mortality rate that varies between 10% and 50%, depending on the amount of hemorrhage and the presence of further injuries to the brain, chest, and abdomen [1].

The management of patients is determined by their physiological condition, the categorization of their fracture, and any accompanying injuries. Surgical stabilization is recommended for unstable injury patterns when nonsurgical therapy is unsuccessful. The ideal timing for definitive fixation is not established; nevertheless, it is advisable to perform early stabilization [2].

Despite the gold standard method of open reduction and internal fixation in the management of pelvic injuries, it is often accompanied by a significant rate of surgical complications and prolonged operative time. Conversely, the external fixator is frequently employed as the primary and impermanent method for stabilizing anterior pelvic ring injuries, particularly in urgent circumstances [3]. External fixators have the advantages of being rapidly placed, effectively fixing the compromised pelvic ring and reducing hemorrhage within the pelvic cavity. Yet, wound infection, loosening of the fixator, and impingement on the skin, as restrictions of patients’ daily activities, such as sitting, rolling over, and sexual intercourse, are the clinical complications reported with external fixators that limit its use [4].

Minimally invasive techniques have gained popularity in managing pelvic injuries due to their lower surgical morbidity incidence [5]. A subcutaneous pelvic internal fixator (INFIX) has been proposed as a comparable alternative to external fixation for the anterior pelvic ring [6]. The procedure entails the placement of spinal pedicle screws in the anterior part of the pelvis (supra-acetabular entry point) and the insertion of a connecting rod beneath the patient’s skin [7].

The subcutaneous pelvic internal fixator demonstrated greater rigidity than the conventional external fixator, and simultaneously, it decreased the infection rate and nursing care because of the elimination of the open pin tracts, which added advantages to this procedure [8].

The objective of this study was to compare the clinical outcomes in patients with unstable pelvic ring fractures treated with INFIX versus plate fixation.

## Patients and methods

### Ethical considerations

This study was approved by the institutional review board (IRB) of the local ethical committee of our insituate (NO. 1107/03/2024), and written informed consent was taken from all patients before the procedure.

### Patients data

Sixty patients who had unstable pelvic ring injuries presented to the emergency department of our hospital during the period from January 2020 to January 2024.

### Group allocation

The patients were randomly divided into two groups; 30 were treated with INFIX, and the other 30 were treated with open plating. The selection of the fixation method depended on pseudo-randomization ( one case treated with INFIX and the next by open plating).

### Inclusion and exclusion criteria

Our inclusion criteria were adult patients with open book fracture pelvis with an indication for anterior fixation with or without unilateral posterior vertical shear injury (B1-B2- C1), windswept-type injury (LC3 -or 61 B3.2), and few cases of lateral compression injury (LC2, or B2.2 or B2.3).

Patients with open fractures with contaminated wounds, combined visceral injuries requiring laparotomy, pathological fractures, skeletally immature patients < 18 years, and associated acetabular fractures were excluded from the study.

### Preoperative assessment

All patients were subjected to the following:

#### History taking

Including patients’ name, age, sex, date of admission, previous pelvic surgery, special habits, mechanism of injury, and presence of associated chronic illnesses.

#### Physical examination

Full clinical examination, including general examination for any associated injuries. Vital signs (blood pressure, temperature, heart rate, respiratory rate) were recorded. We followed the advanced traumatic life support (ATLS) protocol in the initial assessment of all patients. Injury severity score (ISC), which provides an overall score for patients with multiple injuries, was also used in preoperative assessment. Neurological assessment, especially in sacral fracture, vascular assessment, and abdominal \ urological assessment (catheterization is mandatory) were also performed.

#### Radiological assessment

Plain x-ray (AP view, pelvic inlet view, pelvic outlet view) and (judet view to exclude acetabular fracture). Skeletal survey; chest, spine, and extremities x-ray to detect associated injuries. Multi-detector computed tomography (MDCT) was used in all patients for more analysis of fracture pattern, to detect side of fracture of both anterior and posterior ring, and to determine sacral dimorphism if present. Fracture patterns were classified according to Young and Burgess classification [9] and Tile classification [10]. Abdominal ultrasound and duplex were done to exclude visceral and vascular injuries.

**Fig. 1:**
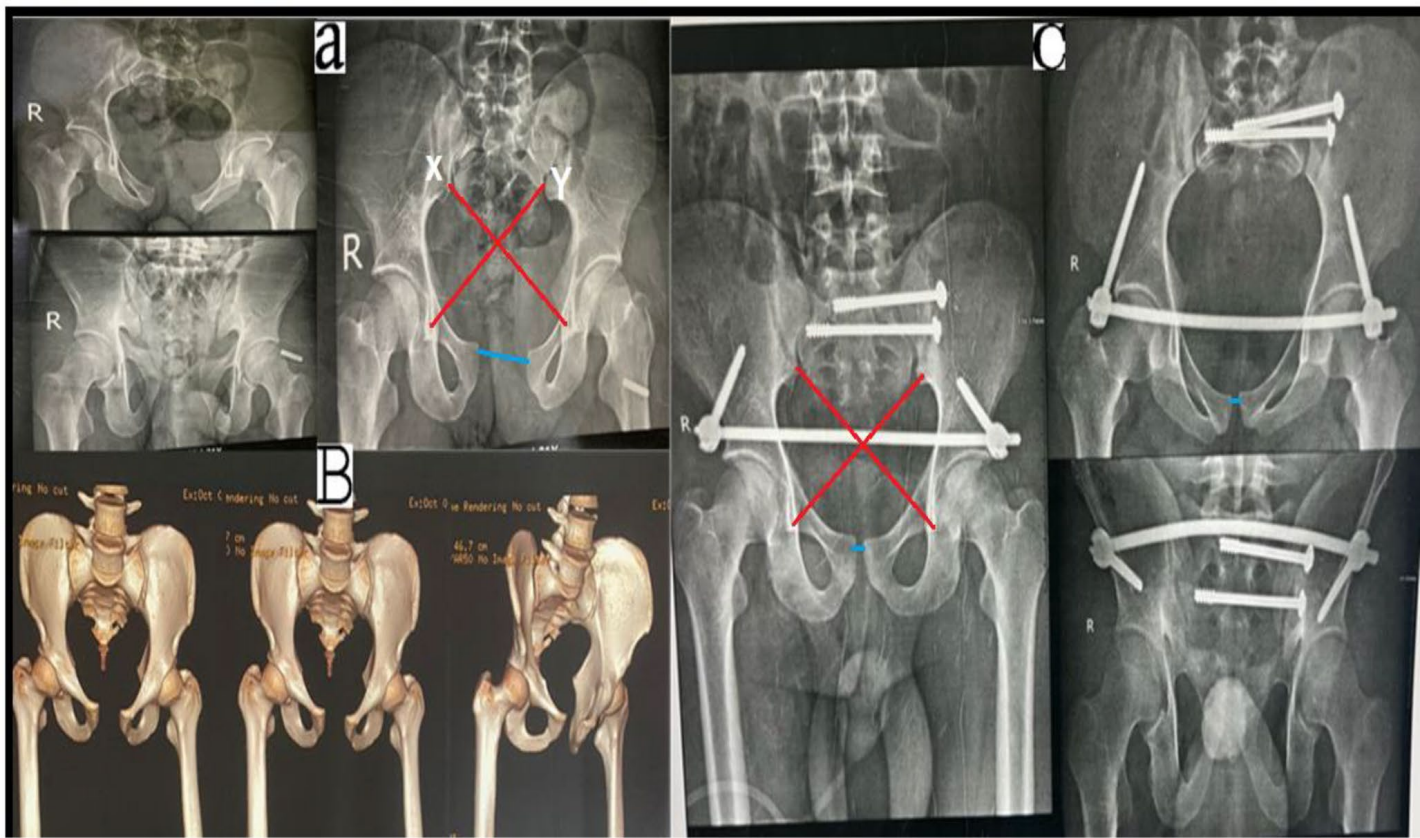
Measurement of pelvic deformity index (PDI) and pubic symphyseal width (PSW) in a 27-year-old male with AP compression type 2 fracture treated by anterior pelvic INFIX. **(A)** Preoperative AP film. **(B)**: Pre-operative MDCT. **(C)**: Postoperative AP film. X or Y is the diagonal length from the inferior SI joint (iliac side) to the inferior aspect of the teardrop on an AP film. PDI = absolute (X − Y)/(X + Y)

Pelvic deformity index and symphyseal pubic widening were used to evaluate the reductions of the pelvic ring. The initial AP pelvis X-ray before applying a pelvic binder, if possible, after fixation, and after hardware removal of the INFIX were done to measure symphyseal diastasis. The cross-method described by Keshishyan [11] and later modified by Lefaivre [12] was used to calculate the pelvic asymmetry value and PDI (Fig. 1).

Preoperative fitness was done for all cases through routine laboratory investigations, including complete blood picture, blood urea and creatinine, bleeding and prothrombin time, and fasting blood sugar.

### Operative procedure

Spinal anesthesia was used for all patients except patients with associated injuries; general anesthesia was used. The supine position was used on a radiolucent operating table under fluoroscopic guidance in all patients. Povidone-iodine, a 7.5% solution, sterilized the surgical field from the umbilical region to the mid-thigh area.

In all patients who developed posterior pelvic ring fracture, the posterior fixation was initially done. In C1 and C2 fractures based on Tile grading, surgical reduction and fixation of an iliac wing fracture using a plate was performed through the lateral window of the ilioinguinal or posterior oblique sacroiliac joint approach according to geometry and proximity to the sacroiliac joint. Sacral fractures or sacroiliac joint disruption were stabilized with minimally invasive techniques by one or two cannulated screws before anterior pelvic ring fixation.

After posterior ring stabilization, anterior ring fixation was then performed either by:

#### Anterior minimal invasive internal fixator (INFIX) group (Figs. 2 and 3)

**Fig. 2:**
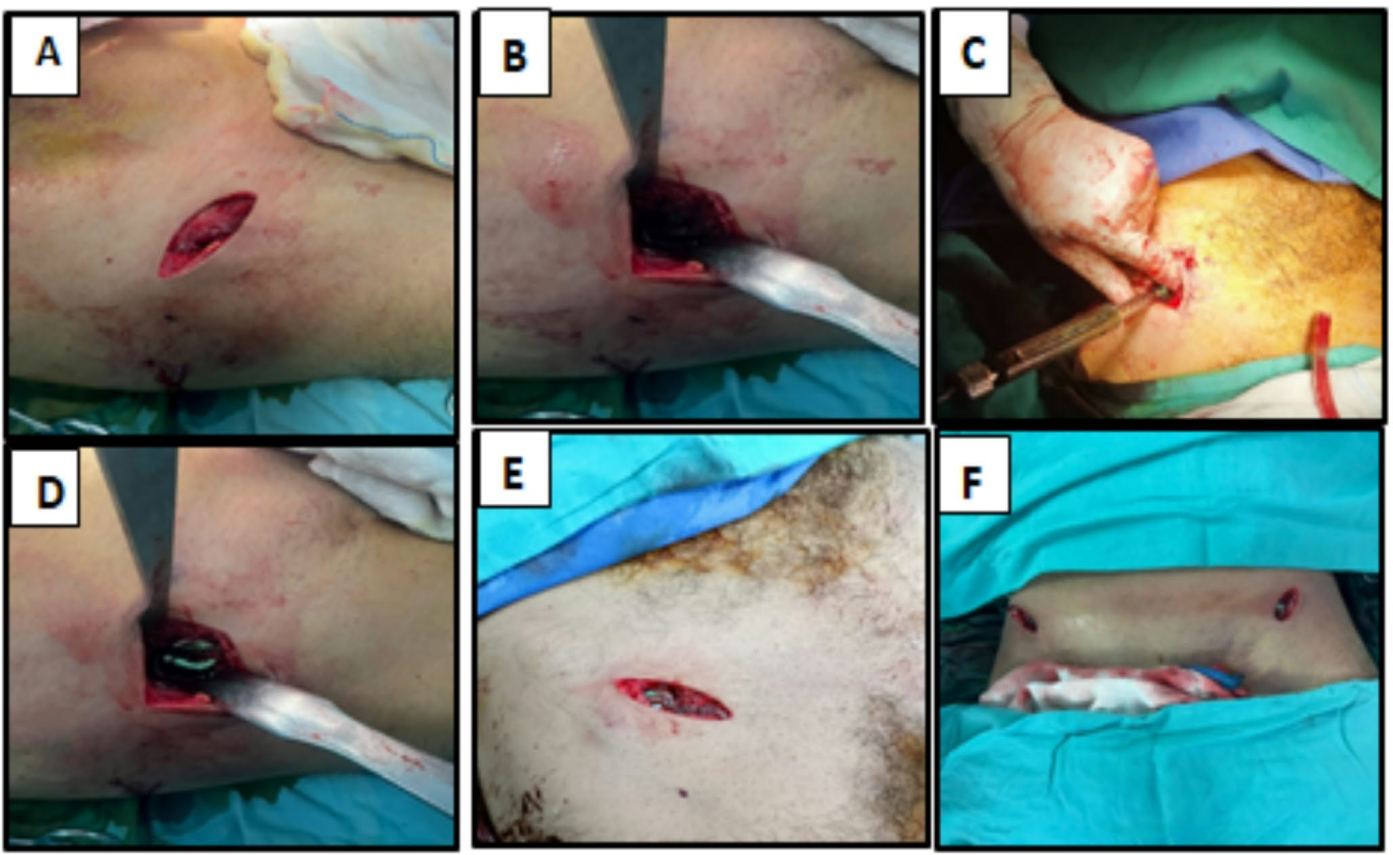
**(A)** Oblique incision centered over AIIS. **(B)** Two Hohmann’s retractors were used to protect the soft tissue. **(C, D)** Pedicle screw head was kept at least 2 cm from the bone surface, (E, F) After completing the procedure

**Fig. 3:**
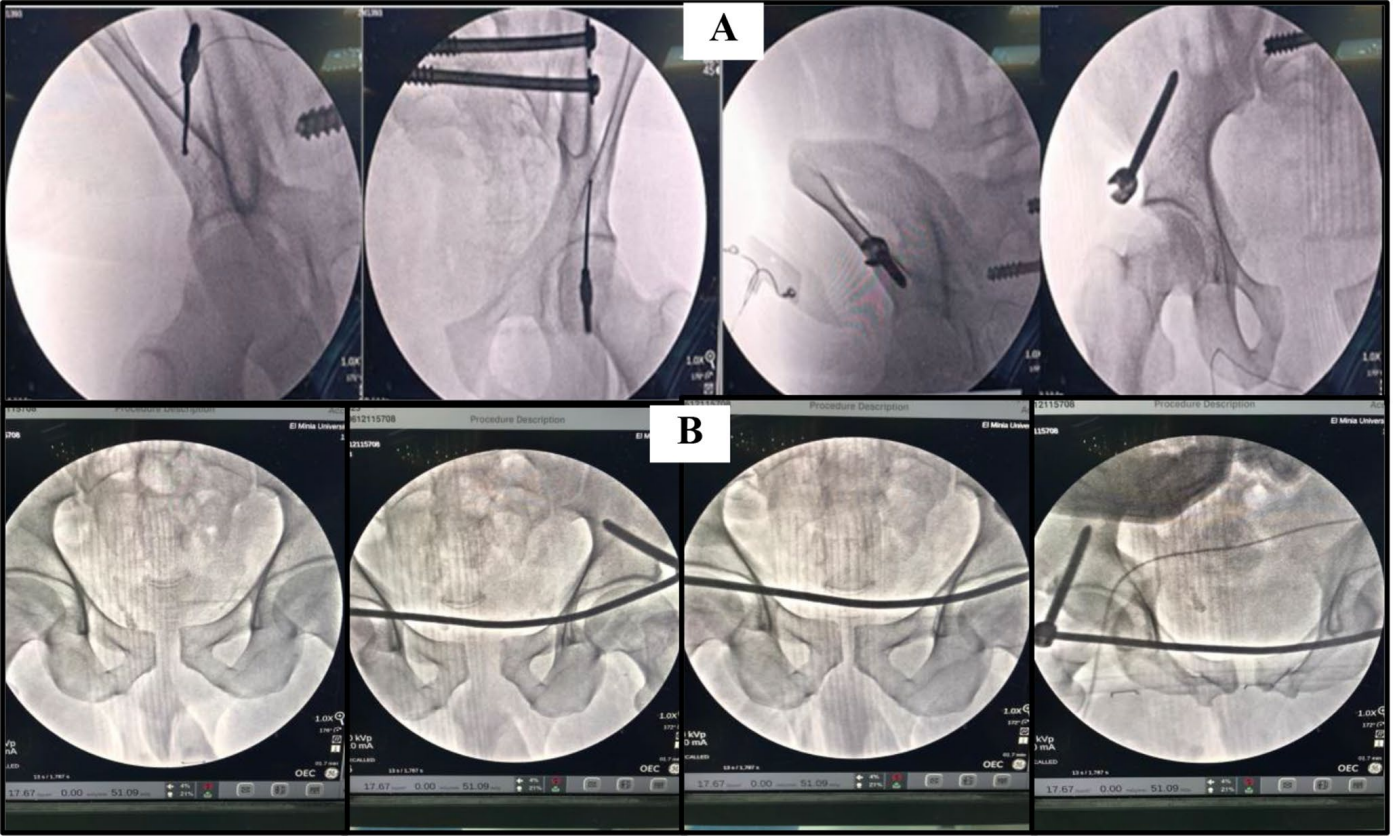
**(A)** screw between inner and outer tables and above the greater sciatic notch. **(B)** showing compression of the anterior pelvic ring

Anterior ring fixation was performed through a 2–3 cm oblique incision centered over the anterior inferior iliac spine (AIIS). Gently, subcutaneous blunt dissection was carried out to avoid the injury of the lateral femoral cutaneous nerve. The intermuscular plane was bluntly developed among the tensor fascia lata and sartorius to expose the AIIS aided by two Hohmann retractors to preserve the soft tissue.

Awl was used to create the entry point between the ilium’s inner and outer bony plates with directions 20 degrees cranio-caudal and 30 degrees medial. A polyaxial pedicle screw (7-mm diameter, 70-mm long) was inserted from the entrance direction of the screw and confirmed with oblique iliac, obturator, and lateral pelvic views to ensure the position of the screw between inner and outer tables (iliac triangle) and above the greater sciatic notch.

The head of the pedicle screw was maintained approximately 2 cm from the bone surface to prevent the compression of the visceral or vascular tissue following putting the connecting rod. The same steps were conducted at the contralateral side of AIIS, followed by a subcutaneous tunnel created from one AIIS incision to the contralateral side traversing the deep fascia. Through this created tunnel, a curved titanium rod was inserted just deep into the sartorius and superficial to the rectus abdominis to connect the bilateral pedicle screws.

After reducing the fracture by indirect mechanisms, either manually or by a compressor, the reduction was checked under the image guide, and the rod was fixed with screws. After that, sufficient space for two overlapping fingers was created between the rod and bone.

#### In the plate group (Fig. 4)

**Fig. 4:**
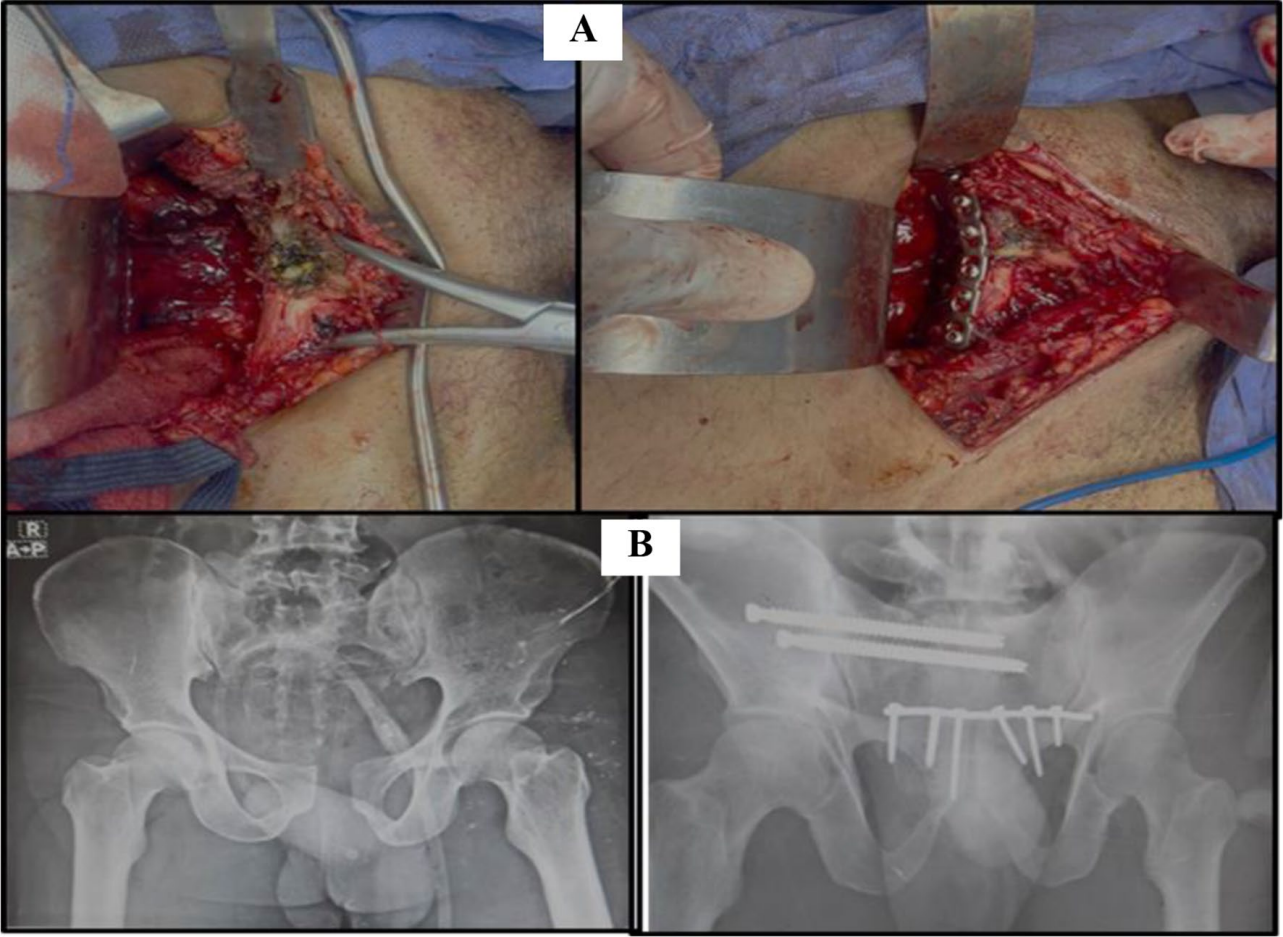
**(A)** Reduction of the diastasis and application of pre-bending reconstructive plate notch. **(B)** X-ray before and after symphyseal plating

Pfannenstiel approach was utilized to place the anterior plate. Preventing iatrogenic bladder injury during the approach was necessary, so a urinary catheter was inserted to decompress the bladder. A 10–15 cm curvilinear or straight incision is centered 2 cm superior to the symphysis pubis and superior pubic ramus. The linea alba was split longitudinally. Then, retract the rectus abdominis muscles laterally without sharp dissection. Direct palpation of the inflated urinary catheter balloon helps to identify the bladder. A laparotomy sponge and wide malleable retractor mobilize and protect the bladder and the peritoneum.

Subperiosteal dissection was extended to expose the anterior and/or posterior surfaces of the pubic body. Laterally, the dissection can be extended as far as the iliopectineal eminence, then a pointed reduction forceps was put over the pubic tubercle or into the obturator foramen to reduce diastasis. A pre-bent reconstructive plate (3.5 mm) was placed at the medial side of the superior pubic rami to fix the symphyseal fragments.

### Postoperative management and follow-up regimen

Intravenous antibiotic injections were administered for three days, and oral forms continued until stitches, analgesics, and oral anticoagulants were removed in some cases (non-ambulatory). The stitches were removed after two weeks, postoperative six weeks of restricted weight bearing followed by progressive partial mobilization protected by crutches. If the fracture healing was established in radiographs, usually after 8–12 weeks, full weight-bearing was permitted. The INFIX was removed three months after insertion or once fracture union occurred.

The patients were clinically assessed by the Majeed rating scale and radiologically at 1, 3, 6, 12, and 18 months at final follow-up visits.

Plain radiographs were done for all patients using three standard views (anteroposterior, inlet, and outlet). We used the Matta/Tornetta [13] and the Lefaivre [12] criteria to evaluate fracture reduction, vertical displacement, and pelvic symmetry based on radiographs and MDCT images.

Fracture reduction was rated as excellent (< 5 mm), good (5–10 mm), fair (11–20 mm), and poor (> 20 mm).

Distances of the rod to bone and the head of a screw to AIIS were calculated and measured on the postoperative MDCT in each case.

### Statistical analysis

The computer program Statistical Package for Social Science (SPSS) version 25 was utilized for data analysis after being tabulated. Descriptive statistics were computed as mean and standard deviation (± SD) for quantitative data; and frequency and distribution for qualitative data.

The significance of difference was tested using one of the following tests after the Kolmogorov–Smirnov normality test to establish their non-normality. Student’s T-test was utilized to compare the means of quantitative parametric data between the two groups. Mann-Whitney’s (U) test was employed to ascertain the statistical significance of the difference in non-parametric data among both groups. The chi-square test was used to evaluate the association between two qualitative variables. Fisher’s exact test/ or Monte Carlo correction assessed the association between qualitative variables if the expected count is below 5 in more than 20% of cells.

P value < 0.05 was considered statistically significant. P value < 0.01 was considered highly important in all analyses.

## Results

A total of 60 patients with unstable pelvic ring injuries were involved in our study, with 38 (63.3%) males and 22 (36.7%) females. Their mean age was 33.57 ± 11.03 years. The patients were randomly classified into two groups: 30 patients were managed by INFIX, and the other 30 patients with open plating.

### Demographic and clinical characteristics

Both groups were comparable in terms of age and gender. The median age in the INFIX group was 34.5 years, with 53.3% being males; in the plate group, the median age was 32.5 years, with 73.3% males, with no significant difference between the two groups (*P* > 0.05). In addition, there is no significant difference between the two groups in terms of comorbidities, mechanism of injury, associated injury, ISS, and Young-burgess classification (*P* > 0.05) (Table 1).

**Table 1:** Demographic and clinical characteristics of the study groups

		INFIX Group (*N* = 30)		Plate Group (*N* = 30)		*P* value
		*N*	%	*N*	%	
Gender	**Male**	16	53.3%	22	73.3%	0.108^‡^
	**Female**	14	46.7%	8	26.7%	
Age (years)	**Mean ± SD** **Median (IQR)** **Range**	34.67 ± 12.6 34.5 (22–44) 19–55		32.47 ± 9.3 32.5 (23–40) 19–48		0.583^╪^
Comorbidities	**No**	22	73.3%	26	86.7%	0.548^‡^
	**Asthmatic**	1	3.3%	0	0.0%	
	**DM**	4	13.3%	2	6.7%	
	**HTN**	3	10.0%	2	6.7%	
Fracture side	**Ant. both**	2	6.7%	0	0.0%	0.07^‡^
	**Post. Lt _ant Lt**	9	30.0%	10	33.3%	
	**Post Lt _ant both**	11	36.7%	20	66.7%	
	**Post Rt _ ant both**	8	26.7%	0	0.0%	
Mechanism of injury	**FFH**	15	50.0%	13	43.3%	0.073^‡^
	**MCA**	12	40.0%	7	23.3%	
	**MVA**	3	10.0%	10	33.3%	
Associated injury	**No**	20	66.7%	21	70.0%	0.120^‡^
	**Bilat calcaneus _ fracture spine**	1	3.3%	0	0.0%	
	**Chest trauma + head trauma**	1	3.3%	0	0.0%	
	**Clavicle fracture**	2	6.7%	0	0.0%	
	**Fracture calcaneus**	0	0.0%	4	13.3%	
	**Fracture femur**	2	6.7%	3	10.0%	
	**Fracture shaft femur + fracture distal tibia**	1	3.3%	0	0.0%	
	**Head trauma**	2	6.7%	0	0.0%	
	**Humerus fracture**	1	3.3%	1	3.3%	
	**Trochanteric fracture**	0	0.0%	1	3.3%	
Injury Severity Score (ISS)	**Mean ± SD** **Median (IQR)** **Range**	18.77 ± 10.56 15.5 (9–21) 9–43		18.60 ± 5.83 18 (14–22) 9–34		0.289^╪^
Tile classification	**B2**	17	56.7%	24	80.0%	0.23^‡^
	**B3**	5	16.7%	0	0.0%	
	**C1**	1	3.3%	0	0.0%	
	**C2**	6	20.0%	6	20.0%	
	**C3**	1	3.3%	0	0.0%	
Young-burgess classification	**AP compression 2**	6	20.0%	9	30.0%	0.087^‡^
	**AP compression 3**	3	10.0%	6	20.0%	
	**Lateral compression 2**	7	23.3%	9	30.0%	
	**Lateral compression 3**	6	20.0%	0	0.0%	
	**vertical shear**	8	26.7%	6	20.0%	
Time to surgery (days)	**Mean ± SD** **Median (IQR)** **Range**	3.33 ± 1.47 3 (2–4) 2–9		4.73 ± 2.48 4.5 (3–7) 1–9		**0.031** ^**╪**^

DM: diabetes mellitus; HTN: hypertension; FFH: fall from height; MCA: motor car accident; MVA: motor vehicle accident

^╪^ Mann- Whitney U test, ^‡^ Chi-square test,

Fracture in the posterior left and anterior right & left sides was found in 36.7% of patients in the INFIX group and 66.7% in the plate group, with an insignificant difference between the two groups (*P* = 0.07). Tile classification showed an insignificant difference between the two groups (*P* = 0.23) as in the INFIX group, 56.7% of patients were classified as B2, 16.7% as B3, 3.3% as C1, 20% as C2, and 3.3% as C3 while in plate group, 80% patients were classified as B2 and 20% as C2 (Table 1).

Young-burgess classification showed that vertical shear is the most common in the INFIX group. At the same time, AP compression 2 (30%) and lateral compression 2 (30%) were the most popular patterns of trauma in the plate group, with insignificant differences among both groups (Table 1).

The time to surgery was significantly lower in the INFIX group (median of 3 days) compared to the plate group (median of 4.5 days) (*P* = 0.031) (Table 1).

### Perioperative parameters

**Chart 1:**
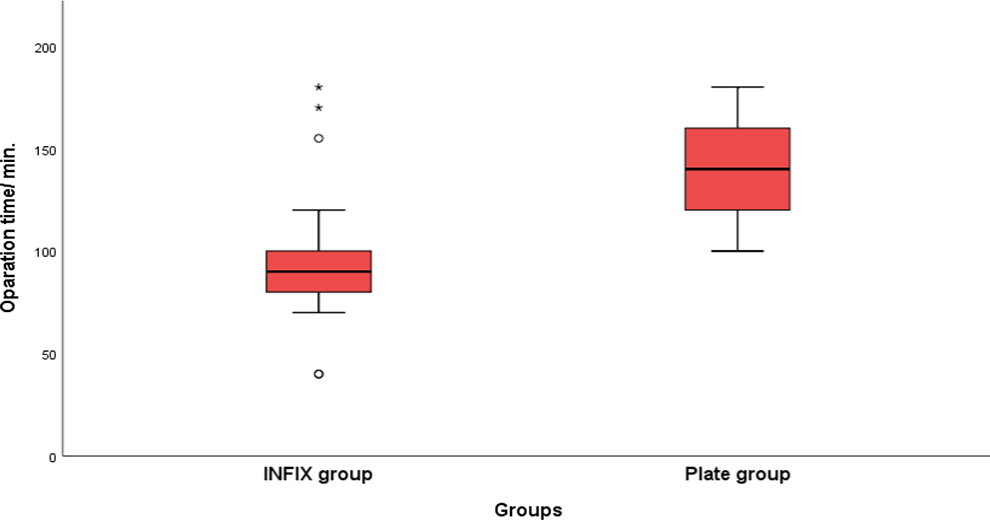
Comparison between the two groups regarding operation time

Table (2) illustrates perioperative data regarding ICU admission; one patient in the INFIX group and four patients in the plate group needed ICU with no significant difference between the two groups (*p* > 0.05). Furthermore, the median time of anterior pelvic ring procedure was significantly lower in the INFIX group compared to the plate group (INFIX group: 40 min and plate group: 65 min, *P* < 0.001). The median whole operative time (Chart 1) and blood loss were significantly lower in the INFIX group than in the plate group (*P* < 0.001).

**Chart 2:**
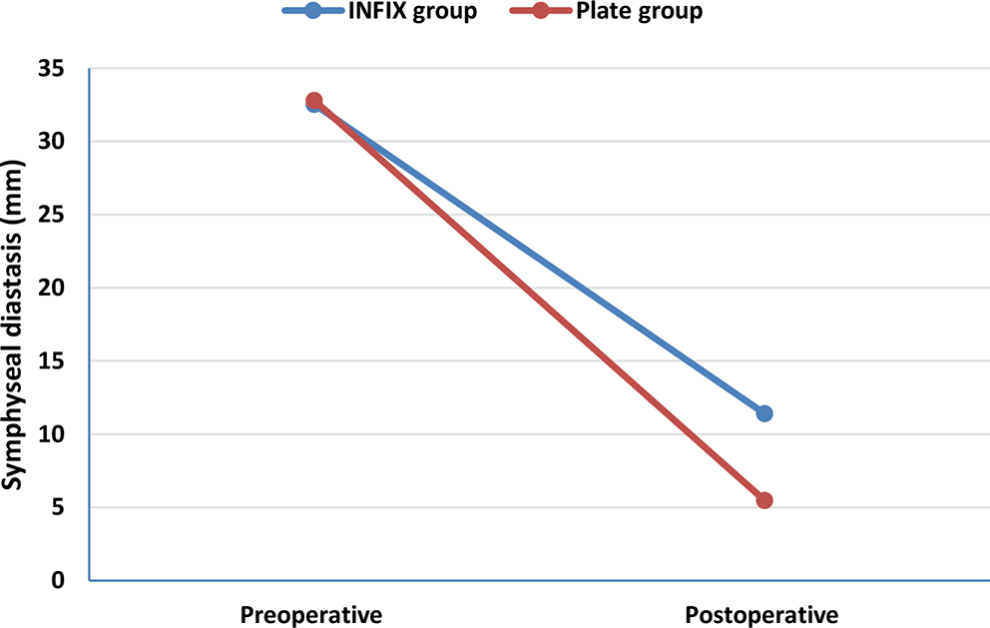
Comparison between the two groups regarding symphyseal diastasis

The hospital stay was significantly shorter in the INFIX group (median of two days) than in the plate group (median of 5 days)(Chart 2). The median implant removal time in the INFIX group was three months, ranging from 1.5 to 4 months. The mean rod-to-bone distance was 21.78 ± 1.74 ml (Table 2).

**Table 2:** Perioperative data of the study groups

		INFIX Group (*N* = 30)		Plate Group (*N* = 30)		*P* value	
		*N*	%	*N*	%		
Need for ICU	**Need ICU**	1	3.3%	4	13.3%	0.353^‡^	
	**No need**	29	96.7%	26	86.7%		
Union time (weeks)	**Mean ± SD** **Median (IQR)** **Range**	13.37 ± 1.61 13 (12–14) 12–18		17.90 ± 3.57 14 (13–15) 12–24		0.069^╪^	
Follow-up (months)	**Mean ± SD** **Median (IQR)** **Range**	18.30 ± 3.24 18 (15–20) 14–24		15.53 ± 3.53 14 (13–18) 13–24		0.08	
Majeed score at the final follow-up.	**Poor**	3	10.0%	3	10.0%	> 0.999^‡^	
	**Fair**	3	10.0%	4	13.3%		
	**Good**	12	40.0%	12	40.0%		
	**Excellent**	12	40.0%	11	36.7%		
Matta score at the final follow-up	**Fair**	5	16.7%	6	20.0%	0.148^‡^	
	**Good**	5	16.7%	11	36.7%		
	**Excellent**	20	66.7%	13	43.3%		
Time of anterior ring procedure (min.)	**Mean ± SD** **Median (IQR)** **Range**	38.0 ± 5.66 40 (35–40) 30–50		63.5 ± 7.21 65 (60–70) 50–75		**< 0.001** ^**╪**^	
Whole Operative time (min.)	**Mean ± SD** **Median (IQR)** **Range**	94.5 ± 30.12 90 (80–100) 40–180		141.33 ± 21.93 140 (120–160) 100–180		**< 0.001** ^**╪**^	
Blood loss (ml)	**Mean ± SD** **Median (IQR)** **Range**	488.67 ± 527.02 200 (150–600) 60- 2000		1263.33 ± 475.78 1200 (1000–1500) 500–2000		**< 0.001** ^**╪**^	
Hospital stay (days)	**Mean ± SD** **Median (IQR)** **Range**	1.87 ± 0.94 2 (1–2) 1–4		4.97 ± 1.38 5 (4–6) 3–7		**< 0.001** ^**╪**^	
Post ring fixation	**No**	2	6.7%	2	6.7%	0.051^‡^	
	**1 Screw**	0	0.0%	5	16.7%		
	**2 Screws**	25	83.3%	18	60.0%		
	**2 Plates**	1	3.3%	0	0.0%		
	**2 Plates and Screw**	2	6.7%	5	16.7%		
Preop. symphyseal diastasis (mm)	**Mean ± SD** **Median (IQR)** **Range**	32.53 ± 10.75 31(23–40) 18–55		32.80 ± 11.38 30 (26–42) 13–54		0.629^ג^	
Postop. symphyseal diastasis (mm)	**Mean ± SD** **Median (IQR)** **Range**	11.40 ± 3.76 12 (8–13) 6–19		5.47 ± 2.03 5 (4–7) 3–10		**< 0.001** ^**ג**^	
P-value between pre & post op.^♦^		**< 0.001**		**< 0.001**			

ICU: intensive care unit

^╪^ Mann- Whitney U test, ^‡^ Chi-square test, ^ג^: Student T test, • Paired T-test

The follow-up period was 18.30 ± 3.24 months in the INFIX group and 15.53 ± 3.53 months in the plate group, with non-significant differences between both groups. The union time had a median of 13 weeks in the INFIX group and 14 weeks in the plate group (*P* > 0.05) (Table 2).

Posterior pelvic ring fixation was performed using two ilio-sacral screws fixation in 83.3% of patients, two plates in 3.3% of patients, and two screws combined with two plates in 6.7% of patients in the INFIX group. Whereas in the plate group, one screw fixation, two screws fixation, and two screws combined with two plates were utilized for posterior pelvic ring fracture in 16.7%, 60%, and 16.7% patients, respectively, with the non-significant difference among both groups (*P* > 0.05).

### Functional and radiological outcomes

The patients were followed up postoperatively by Majeed and Matta scores. Majeed score was excellent and good in 80% of patients treated with INFIX and 76% of patients treated with plating, with no significant difference between the two groups (*P* > 0.05). As for Matta’s score, excellent and good scores were in 83% and 80% of cases in the INFIX and plate groups, respectively, with insignificant differences between the two groups.

**Chart 3:**
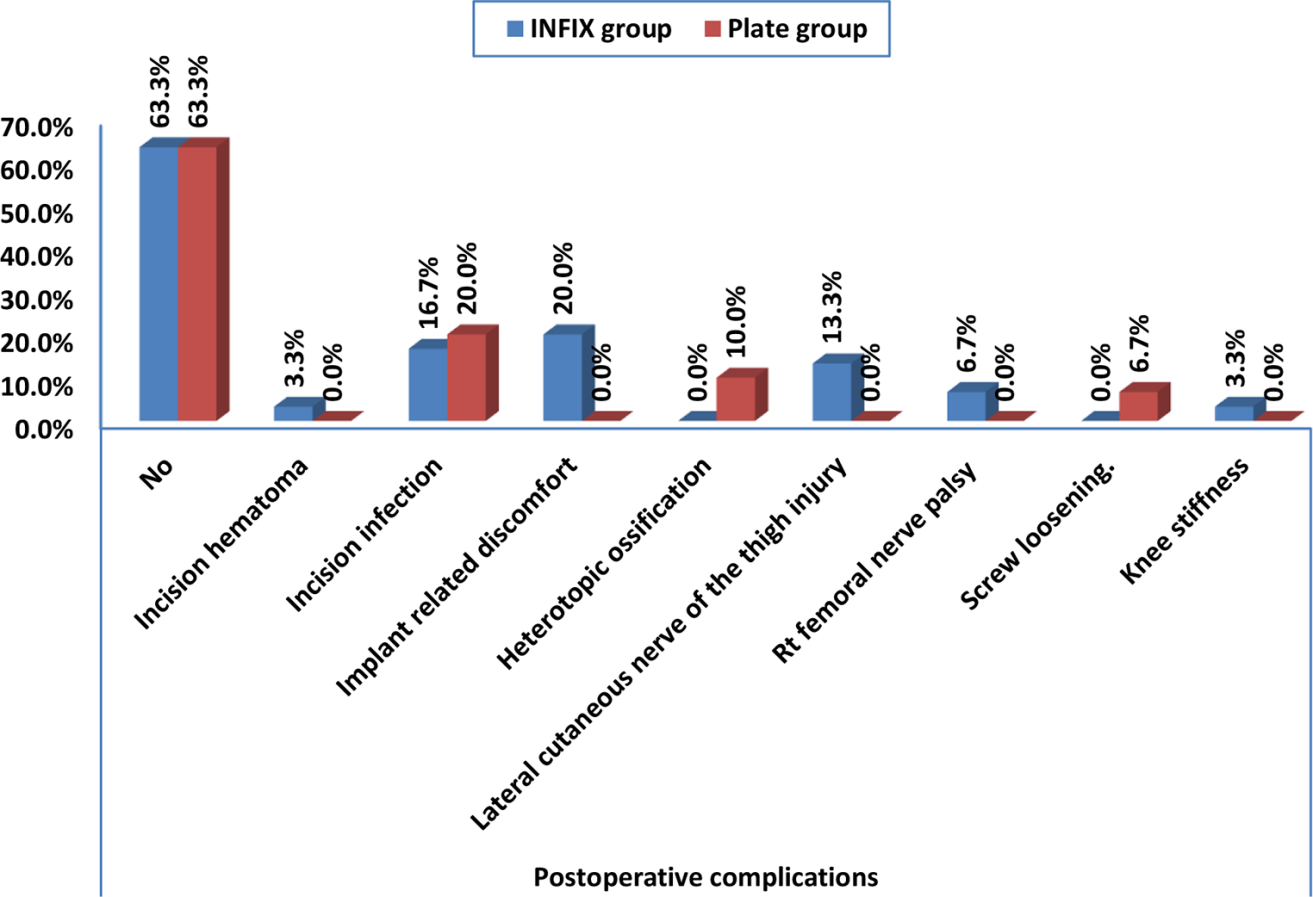
Comparison between the two groups regarding complications

Radiological symphyseal diastasis (Table 2 and Chart 3) was assessed preoperatively and postoperatively. The INFIX group reported significantly wider symphyseal diastasis postoperatively than the plate group (*P* < 0.001), with no significant difference between them preoperatively (*p* > 0.05). Significant changes (decrease) in radiological symphyseal diastasis were found between pre and postoperative views in both groups (*P* < 0.001).

### Postoperative complications

**Chart 4:**
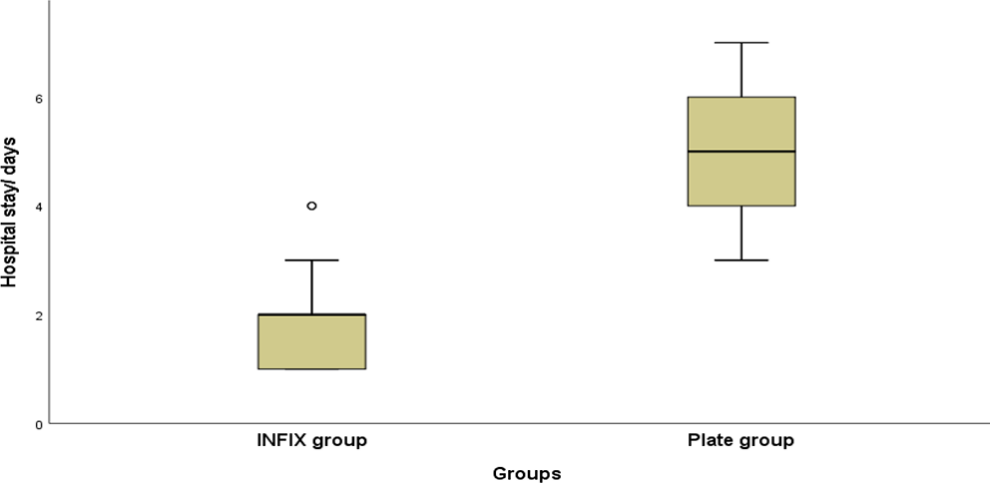
Comparison between the two groups regarding hospital stay

Postoperative complications occurred in 11 (36.7%) patients in both groups. The INFIX group showed a significant increase in developing implant-related discomfort compared to the plate group (*P* = 0.031). Other complications reported were incision hematoma due to anticoagulant (1 case in INFIX group), incision infection (5 cases in INFIX versus 6 cases in plate group), heterotopic ossification (3 in plate group), lateral cutaneous nerve of the thigh injury (4 cases in INFIX), right femoral nerve palsy (2 cases in INFIX group), screw loosening (2 cases in plate group) and knee stiffness (1 case in plate group) (Table 3, Chart 4).

**Table 3:** Postoperative data of the study groups

		INFIX Group (*N* = 30)		Plate Group (*N* = 30)		*P* value
		*N*	%	*N*	%	
Postop. Complications	**No**	19	63.3%	19	63.3%	0.789^‡^
	**Incision hematoma due to anticoagulant**	1	3.3%	0	0.0%	> 0.999^‡^
	**Incision infection**	5	16.7%	6	20.0%	> 0.999^‡^
	**Implant related discomfort**	6	20.0%	0	0.0%	0.031^‡^
	**Heterotopic ossification**	0	0.0%	3	10.0%	0.237^‡^
	**Lateral cutaneous nerve of the thigh injury**	4	13.3%	0	0.0%	0.112^‡^
	**Rt femoral nerve palsy**	2	6.7%	0	0.0%	0.492^‡^
	**Screw loosening.**	0	0.0%	2	6.7%	0.492^‡^
	**Knee stiffness**	1	3.3%	0	0.0%	> 0.999^‡^
Implant removal time (months)	**Mean ± SD** **Median (IQR)** **Range**	2.92 ± 0.67 3 (2.5-3) 1.5- 4				
rod to bone (ml)	**Mean ± SD** **Median (IQR)** **Range**	21.78 ± 1.74 22 (20-22.9) 18–25				

^╪^ Mann- Whitney U test, ^‡^ Chi-square test, ^ג^: Student T-test

* More than one complication may be found in the same patient

## Discussion

The pelvic ring consists of two innominate bones and the sacrum. The anterior pelvic ring comprises bilateral pubic rami joined by the pubic symphysis. The sacrum and two innominate bones connected at the sacroiliac joints by ligaments form the posterior ring [14].

High-energy pelvic injuries are a relatively uncommon type of injury, accounting for 2–8% of all fractures in the human body. They are typically induced by significant, life-threatening external forces like those experienced in motorbike accidents or falls from a considerable height. Unstable pelvic injuries that result from this high-energy trauma are commonly associated with young patients between 15 and 30 years [15].

Anterior ring anchoring addresses unstable posterior ring fixation, reinforces the pelvic ring, or treats a standalone straddle fracture. Anterior pelvic ring stabilization can be accomplished through external or internal fixation techniques. The internal fixation methods include a plate, an antegrade or retrograde screw, a subcutaneous pelvic bridge by a spinal implant, an anterior pelvic internal fixator with supra-acetabular spinal pedicle screws, and the INFIX, or its modifications [16].

INFIX is a newer modality for managing pelvic ring injuries with its compatible outcomes and the external fixators, but it also avoids external fixator complications. INFIX is not yet considered a popular treatment option for anterior pelvic ring injuries amongst pelvic surgeons worldwide [17]. A search of Pubmed about pelvic infix yielded 90 studies since 2012; a few studies (about four) directly compare INFIX versus plate osteosynthesis. Therefore, we aimed in our trauma center to assess INFIX outcomes compared to internal plating for the management of different patterns of unstable pelvic ring injuries.

Regarding demographic data, both groups were comparable in age, with males being predominant in both groups. Comorbidities, mechanism of injury, associated injury, ISS, and Young-burgess classification showed no significant difference between the groups. These results go in line with many types of research that evaluated cases with unstable anterior pelvic ring fractures after treatment with INFIX and plate fixation, as mentioned by Yin et al. [18], Huang et al. [19], and Yu et al. [20].

Tile classification of our patients showed an insignificant difference between the two groups, with B2 being the most common in 56.7% and 80% of the patients in INFIX and plate groups, respectively. Yin et al. [18] study agrees with our results in Tile classification as there was no statistically significant difference between the two groups, with B2 being the most common in both groups.

Concerning perioperative data in our study, we revealed that one patient in the INFIX group and four patients in the plate group needed ICU with no significant difference between the two groups (*P* > 0.05). This was less than what was reported by Ansari et al. [21], who evaluated the outcome of INFIX in anterior pelvic ring fractures and found that 16 patients who had undergone INFIX needed an ICU stay.

Patients treated with INFIX showed significantly lower time of anterior ring procedure, lower whole operative time, and less blood loss than the plate group; these results align with Yin et al. [18], who revealed that the INFIX group demonstrated superiority over the plate group regarding procedure time and blood loss, in the same line Yu et al. [20] showed that the operation time and intraoperative blood loss in INFIX group were significantly less than those in plate group. In the same direction, Ansari et al. [21] revealed less blood loss (125 ml) and short average surgical time (83 min) in patients with pelvic ring fractures managed with INFIX, and this supports our data.

The follow-up period of our patients was up to 18 months, with an insignificant difference between the two groups; this was less than the study of Yin et al. [18], as they mentioned that the median follow-up was 27 months and 23 months in the INFIX group and plate group respectively. The same Huang et al. [19] reported a more extended follow-up period than our study (26 months in the INFIX group and 33 months in the plate group).

Regarding hospital stay, it was significantly shorter in the INFIX group compared to the plate group as it is a minimally invasive procedure; this coincides with Pan et al. [22], who reported the same.

The union time had a median of 13 weeks in the INFIX group and 14 weeks in the plate group (*P* > 0.05). This is compatible with Wu et al. [23], who recorded that all patients treated with INFIX achieved bony union with an average of 13.3 weeks.

Concerning functional and radiological outcomes, the Majeed and Matta scores showed no significant difference between the two groups (*P* > 0.05). Our results are consistent with those of Yin et al. [18] and Huang et al. [19], as both studies revealed that no statistically significant difference was found in the individual items of the Majeed scores among both procedures.

Regarding posterior pelvic ring fixation, the INFIX group reported significantly wider symphyseal diastasis postoperatively than the plate group. A significant decrease in symphyseal diastasis was found between the pre-and postoperative radiographs in each group. These data suggest that open plating achieved a better anatomical reduction than INFIX. Moreover, Vaidya et al. [24] reported that INFIX was inferior to plating at reducing symphyseal widening. On the contrary, Tsai et al. [25] revealed no statistically significant difference between the two groups regarding posterior pelvic ring fixation.

Concerning complications, we reported 11 (36.7%) patients developed postoperative complications in both groups. Both groups had an insignificant difference in the reported complications, except implant-related discomfort, which was significantly more reported in the INFIX group. Our results are consistent with those of Pan et al. [22], who found no statistically significant difference between the two groups regarding postoperative complications.

In contrast with our results, Yu et al. [20] revealed that postoperative complications statistically significantly differed between both groups, as the incidences of lateral femoral cutaneous nerve injury, femoral nerve injury, and heterotopic ossification were considerably higher in the INFIX group than in plate group, while the incidence of incision infection was higher in plate group than in INFIX group. Similarly, Vaidya et al. [24] reported a marked incidence of infection, improper hardware placement or failure, and heterotopic ossification in the plate group compared to the INFIX group.

### Limitations

There are a few notable limitations in our study in the form of a short follow-up period, as it was retrospective. Moreover, this study’s relatively small sample size may not represent all heterogeneous pelvic ring injuries, and this was a single-center study.

Further, multi-center studies are considered. In addition, research to assess the biomechanical stability and the outcomes of titanium elastic nails, percutaneous retrograde suprapubic screws, and newer variations of INFIX (unilateral INFIX, extended unilateral INFIX, and extended bilateral INFIX) is a plan, especially anterior pelvic ring disruption.

## Conclusion

Based on our findings, we conclude that INFIX is a relatively minimally invasive, safe, and easy technique with potential transient complications for managing even complex pelvic ring injuries. The INFIX had several advantages over plating, such as time-saving, less blood loss with a satisfactory bony union, and adequate functional results; however, plating provides better anatomical and radiographic fracture reduction with consideration given due to favorable functional scores in both techniques.

## Data Availability

No datasets were generated or analysed during the current study.

## Abbreviations

INFIX: Anterior Subcutaneous Internal Fixation
ASIS: Anterior Superior Iliac Spine
AIIS: Anterior Inferior Iliac Spine
ISS: Injury Severity Score
MDCT: Multi-Detector Computed Tomography
