# Correction: Anterior minimal invasive internal fixator versus open plating in treatment of unstable pelvic ring injuries

**DOI:** 10.1007/s00402-025-05986-7

**Published:** 2025-07-30

**Authors:** Khaled Omran, Ahmed Nady, Ahmad Hisham Abdelhalim Ali, Mohamed Sayed Khamies

**Affiliations:** Department of Orthopedic Surgery and Traumatology, Minia University Hospital, Faculty Of Medicine, Minia University, Minya, Egypt

**Correctio to: Archives of Orthopaedic and Trauma Surgery (2025) 145:282**

10.1007/s00402-025-05891-z

In the original version of this article, the author’s name ‘Ahmad Hisham Abdelhalim Ali’ was incorrectly written as ‘Ahmed Hisham Abdelhaliem Ali’.

